# Hemostasis using a covered self‐expandable metal stent for pseudoaneurysm bleeding from the perihilar bile duct

**DOI:** 10.1002/deo2.150

**Published:** 2022-06-30

**Authors:** Yu Ishii, Akihiro Nakayama, Kazuo Kikuchi, Kei Nakatani, Kenichi Konda, Daichi Mori, Shigetoshi Nishihara, Shu Oikawa, Tomohiro Nomoto, Tomono Usami, Toshihiro Noguchi, Yuta Mitsui, Hitoshi Yoshida

**Affiliations:** ^1^ Department of Medicine, Division of Gastroenterology Showa University School of Medicine Tokyo Japan; ^2^ Kawasaki Kyodo Hospital Kanagawa Japan

**Keywords:** endoscopic retrograde cholangiopancreatography, hemobilia, interventional radiology, metal stent, pseudoaneurysm

## Abstract

Although there are many reports of hemostasis with covered self‐expandable metal stent (CSEMS) for bleeding from the papilla of Vater and the intrapapillary and distal bile duct, there are rare reports of its use for hemostasis in the perihilar bile duct. We report the case of a patient undergoing supportive care for perihilar cholangiocarcinoma with acute cholecystitis after side‐by‐side placement of uncovered SEMS for perihilar bile duct obstruction. Percutaneous transhepatic gallbladder aspiration was performed upon admission, and hematemesis occurred the next day. Since computed tomography scanning showed a pseudoaneurysm in the right uncovered SEMS, hemostasis by interventional radiology (IVR) was performed thrice for massive bleeding; however, hemostasis could not be achieved. When endoscopic retrograde cholangiopancreatography was performed for scrutiny and treatment of melena and increased hepatobiliary enzyme, the endoscopic visual field could not be secured by bleeding, and changes in hemodynamics were observed; thus, IVR was required, but it was difficult to perform. Since bleeding from the right bile duct was expected, hemostasis was performed using CSEMS. This is the first report of hemostasis performed by placing a covered SEMS for bleeding from a pseudoaneurysm of the intrahepatic bile duct.

## INTRODUCTION

Hemobilia is a disease that causes anemia progression due to bleeding, obstructive jaundice, and acute cholangitis due to coagulation. Although there are many reports of hemostasis with covered self‐expandable metal stent (CSEMS) for bleeding from the papilla of Vater and the intrapapillary and distal bile duct,^1^, ^2^ there are rare reports of its use for hemostasis in the perihilar bile duct. This case is the first report of hemostasis being performed by placing a covered SEMS for bleeding from a pseudoaneurysm of the intrahepatic bile duct.

## CASE REPORT

**FIGURE 1:**
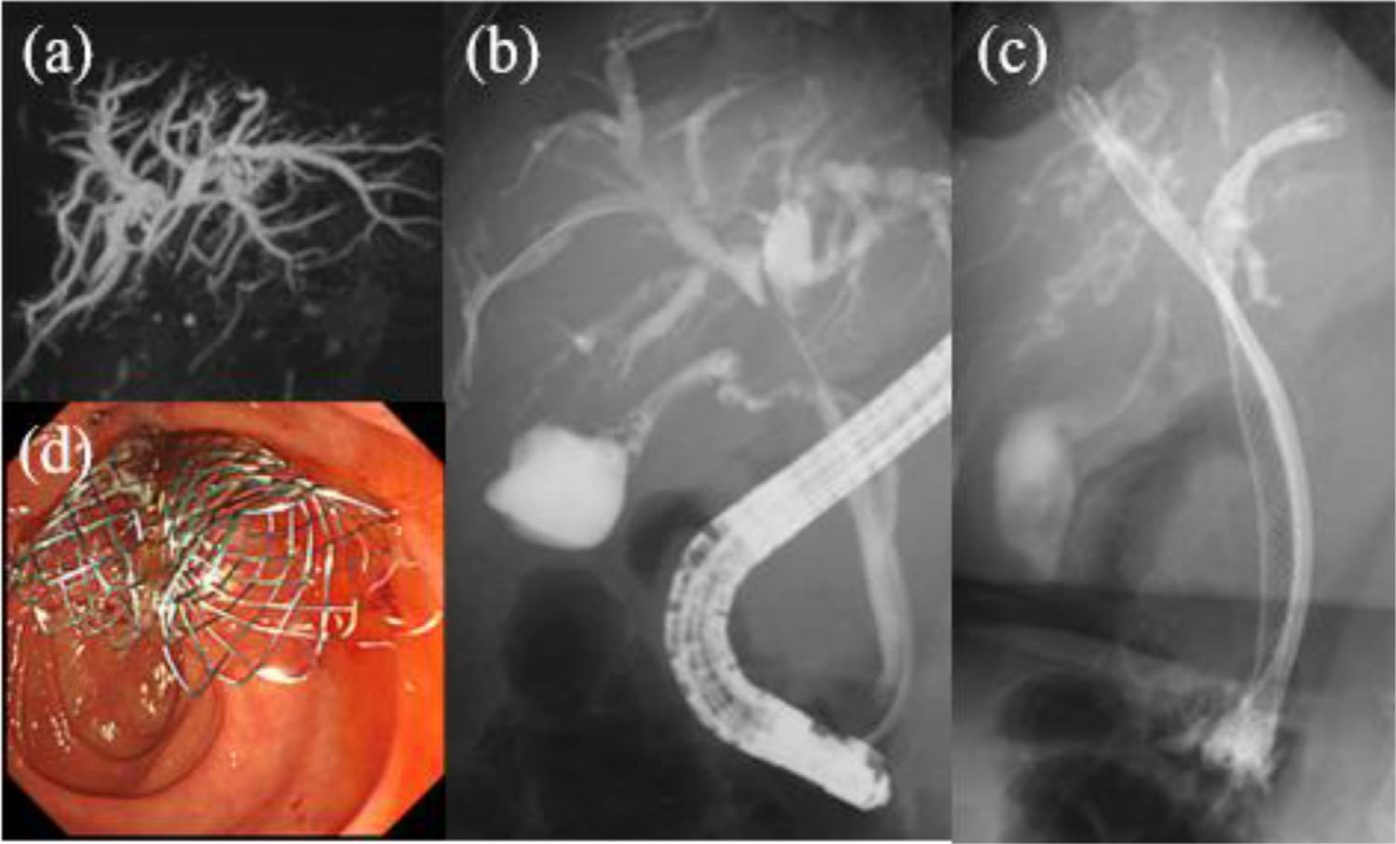
(a) Magnetic resonance cholangiopancreatography finding. (b) ERCP finding before endoscopic SEMS placement. (c) ERCP finding when SEMS was placed side‐by‐side. (d) Endoscopy finding when SEMS was placed side‐by‐side. ERCP, endoscopic retrograde cholangiopancreatography; SEMS, self‐expandable metal stent

**FIGURE 2:**
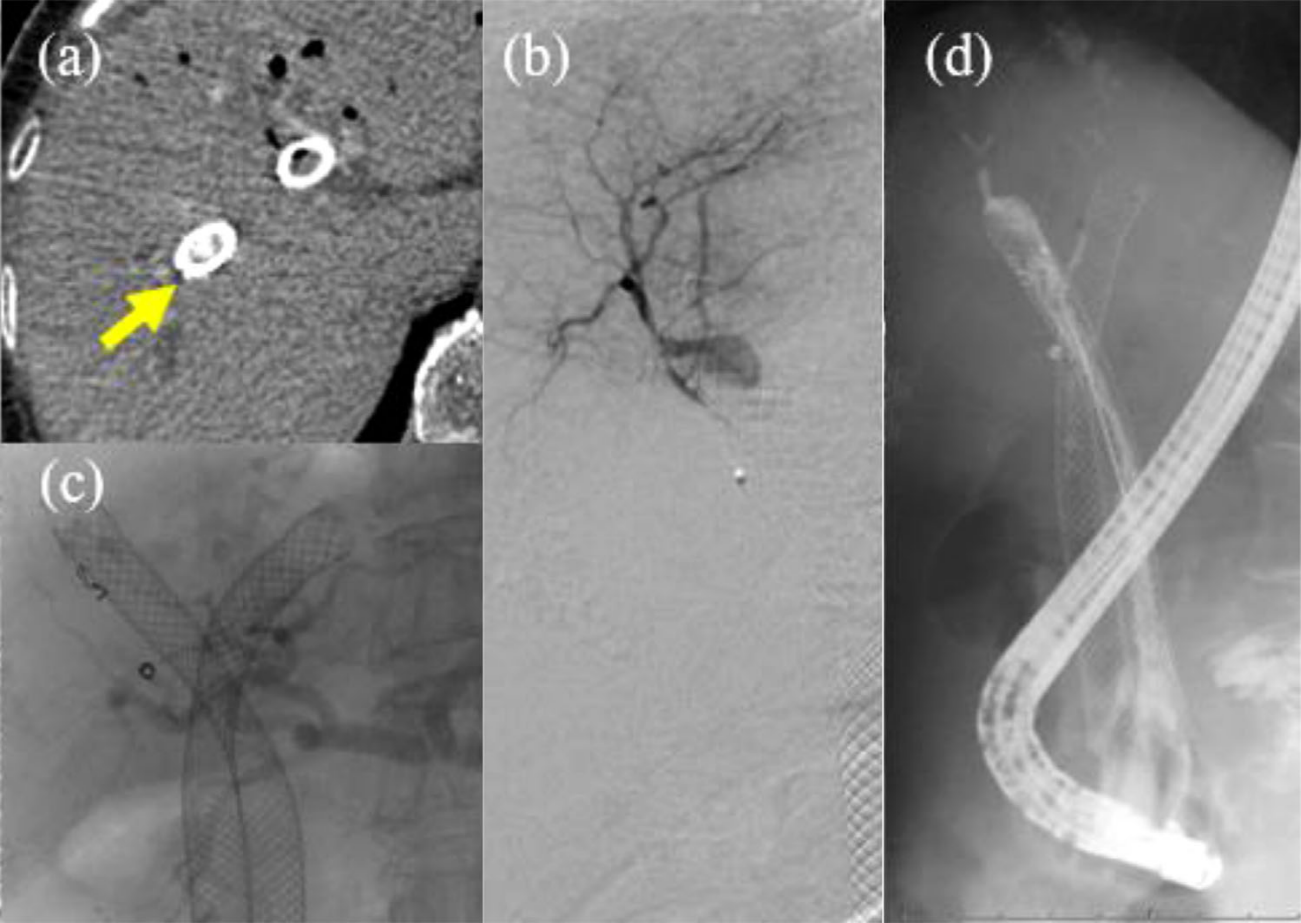
(a) CT scan results showing a pseudoaneurysm in SEMS. The yellow arrow indicates a pseudoaneurysm in SEMS (b) Angiography showing a pseudoaneurysm. (c) Angiography showed A8 of caliber immobility along the upper edge of the stent and pseudoaneurysm in SEMS was noted. Coiling was performed on A8 and the origin of A8 near the pseudoaneurysm. Angiography in the pseudoaneurysm disappeared, but blood flow in A8 remained; therefore, Embosphere was used from the origin of A8. (d) After IVR, ERCP showed a pseudoaneurysm at the upper edge of the right SEMS. CT, computed tomography; SEMS, self‐expandable metal stent. IVR, interventional radiology

**FIGURE 3:**
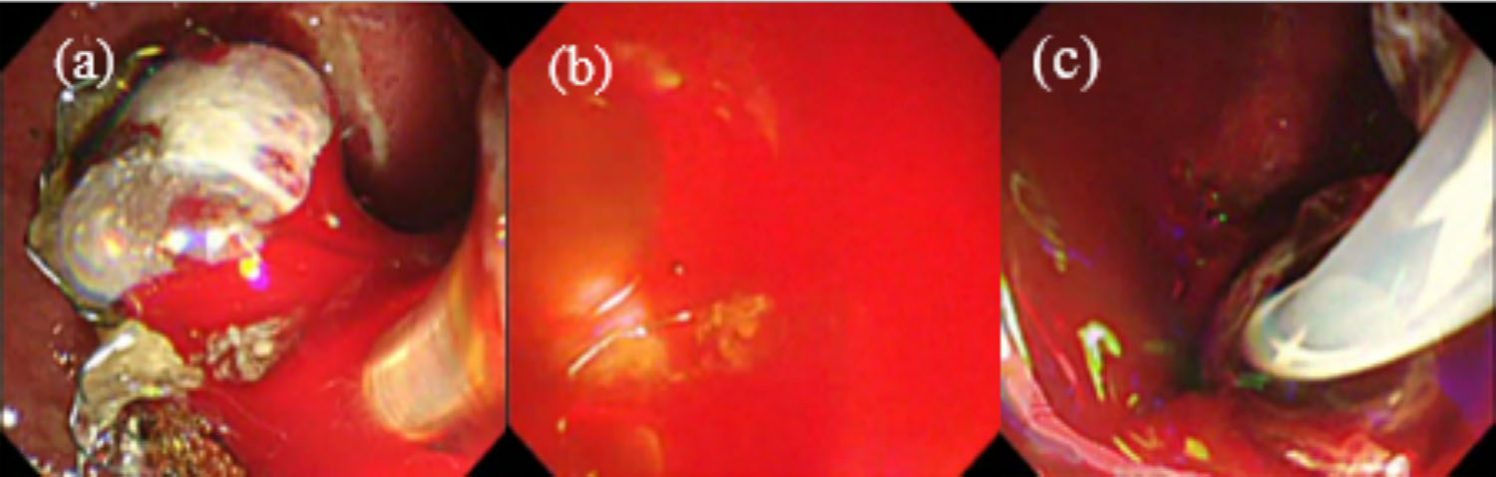
(a) Endoscopic findings before covered self‐expandable metal stent (CSEMS) placement. Blood and embolic material flow into the duodenum. (b) Endoscopic findings before CSEMS placement, wherein it is difficult to secure the field of view. (c) Endoscopic findings after CSEMS placement, wherein the bleeding subsided

**FIGURE 4:**
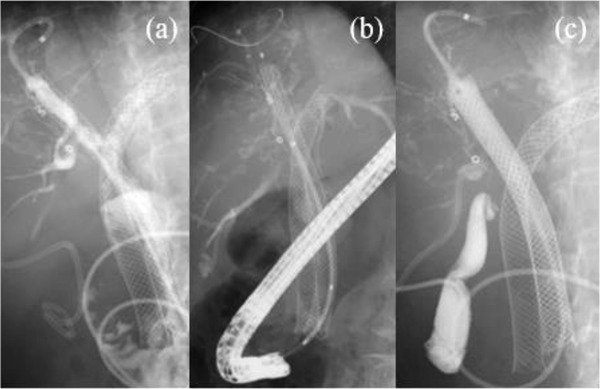
(a) Before CSEMS, cholangiography from endoscopic nasobiliary drainage (ENBD) showed stenosis in the right hepatic duct and disappearance of the pseudoaneurysm was seen in the upper edge of the SEMS. (b) CSEMS was placed from the upper edge of SEMS to control bleeding. (c) Cholangiography findings after CSEMS placement and PTBD. CSEMS, covered self‐expandable metal stent; PTBD, percutaneous transhepatic biliary drainage

The patient was an 88‐year‐old woman who was referred to our hospital for obstructive jaundice. Computed tomography (CT) scanning showed hilar bile duct dilation and stenosis. Endoscopic retrograde cholangiopancreatography (ERCP) showed obstruction from the right hepatic duct to the left hepatic duct (Bithmuth IIIA). Moreover, adenocarcinoma was observed at the bile duct obstruction; thus, a diagnosis of perihilar cholangiocarcinoma was made. We proposed surgery; however, the patient declined and opted for the best supportive care. The patient was discharged after performing side‐by‐side placement of a 10 × 100 mm braided‐type uncovered SEMS (WallFlex Biliary Rx Stent; Boston Scientific, Natick, MA, USA; Figure 1). After 39 days, she consulted a hospital for abdominal pain and fever. Blood test results showed inflammation, such as elevated C‐reactive protein (white blood cell count, 4500 × 10^3^/μl; hemoglobin, 10 g/dl; aspartate aminotransferase, 40 U/L; alanine aminotransferase, 21 U/L; alkaline phosphatase, 406 U/L; γ‐glutamyltransferase, 68 U/L; and C‐reactive protein, 2.57 mg/dl), while CT results showed an enlarged gallbladder. She was hospitalized with a diagnosis of cholecystitis, and percutaneous transhepatic gallbladder aspiration was performed on the same day. Hematemesis occurred on the 2nd day after hospital admission, and blood test results showed the progression of anemia and increased inflammation and hepatobiliary enzyme (white blood cell count, 6500 × 10^3^/μl; hemoglobin, 8.6 g/dl; total bilirubin, 1.8 mg/dl; aspartate aminotransferase, 197 U/L; alanine aminotransferase, 117 U/L; alkaline phosphatase, 1078 U/L; γ‐glutamyltransferase, 296 U/L; and C‐reactive protein, 4.89 mg/dl). Contrast‐enhanced CT with a thinner slice than usual was performed on suspicion of hemobilia. It showed a pseudoaneurysm in the right bile duct stent. Angiography showed A8 of caliber immobility along the upper edge of the stent and pseudoaneurysm in SEMS was noted. Coiling was performed on A8 and the origin of A8 near the pseudoaneurysm. Angiography in the pseudoaneurysm disappeared, but blood flow in A8 remained; therefore, Embosphere was used from the origin of A8. Blood transfusion and endoscopic nasobiliary drainage (ENBD; 6‐Fr) were performed (Figure 2). On the 8th day, melena and bleeding from ENBD were observed, and the source of bleeding was not clear even when interventional radiology (IVR) was performed. A8 was embolized with a Gelpart, and blood was transfused. Percutaneous transhepatic gallbladder drainage (PTGBD) was performed on the 11th day because of recurrent cholecystitis. Due to rebleeding on the 19th day, hemostasis was performed by embolizing A4 with Gelpart and the anterior segment with *N*‐butyl‐2‐cyanoacrylate (NBCA). Blood transfusion and ENBD were also performed. Subsequently, no bleeding was observed. However, fever and hematemesis were observed, and blood examination showed the progression of anemia and increased inflammation and hepatobiliary enzyme on the 31st day. ERCP showed a stenosis of the right hepatic duct on the 32nd day. Biliary loose balloon cleaning for stenosis on the duodenal side of the site where the pseudoaneurysm caused severe bleeding with a poor endoscopic visual field. Changes in the hemodynamics of the patient were observed (blood pressure decreased due to remarkable tachycardia, but only one vital record in ERCP remains. The blood pressure was 145/96 mmHg, and the pulse rate was 107/min). IVR was required; however, no one could perform IVR during that time. Therefore, hemostasis was performed by placing a 10 × 80 mm CSEMS (WallFlex Biliary RX Fully Covered Stent; Boston Scientific Corporation) in the SEMS of the right bile duct. Subsequently, ENBD and blood transfusion were performed (Figures 3 and 4). However, on the 36th day, she developed cholangitis in the posterior segment; thus, percutaneous transhepatic biliary drainage (PTBD) was performed. Subsequently, since there was no bleeding, the patient was informed about CSEMS removal; however, the patient did not want the CSEMS removed because of the long hospitalization and the inability to complete hemostasis by IVR. In addition, PTBD and PTGBD removal was proposed; however, the patient wished to be discharged from the hospital with PTBD and PTGBD indwelling due to the risk of recurrence of acute cholangitis. After discharge, a total of three PTBD exchanges were performed and, the patient was admitted to the palliative care unit one year later.

## DISCUSSION

Hemobilia is a disease that causes the progression of anemia due to bleeding, obstructive jaundice, and acute cholangitis due to coagulation. Bleeding from a pseudoaneurysm often causes shock and is, therefore, a serious disease. The causes of a pseudoaneurysm were inflammation, tumor invasion, iatrogenicity, and trauma. There is also a report on pseudoaneurysms after endoscopic biliary stenting.^3^ In this case, the pseudoaneurysm was in the gallbladder away from the puncture route. Although tumor invasion of the right hepatic artery was observed, it was considered that the main cause of pseudoaneurysm was overdilation by SEMS because an uneven diameter of the artery along the upper edge of the SEMS was observed. IVR is the recommended treatment for a pseudoaneurysm.^4^ Moreover, removal and drainage must be performed for coagulation. It has been reported that the lumen of the stent may be clogged due to blood coagulation.^5^ Furthermore, an ENBD that can be washed is preferred.

Although there are many reports of hemostasis by CSEMS for hemobilia from the papilla of Vater, intrapapillary and distal bile duct, and bleeding after endoscopic sphincterotomy,^1^, ^2^ there is a rare report of its use for bleeding from the perihilar bile duct. Although it was reported that bleeding from the left hepatic duct due to hepatocellular carcinoma of a patient with cirrhosis underwent hemostasis with CSEMS,^6^ this may be because hemostasis by CSEMS in the perihilar bile duct causes contralateral biliary obstruction, which increases the risk of acute cholangitis and obstructive jaundice. Therefore, since there was no bile obstruction, hemostasis with IVR may be better for bleeding from a pseudoaneurysm of the perihilar bile duct if NBCA was not obstructed in the bile duct. However, in cases of changes in hemodynamics due to severe bleeding similar to this one, CSEMS placement may be useful as a temporary technique for hemostasis if IVR is not possible or available in facilities since CSEMS can be removed.^7^ After controlling bleeding, CSEMS removal and hemostasis with IVR must be considered in preparation for the deterioration of hemodynamics and to decrease the risk of acute cholangitis and jaundice. However, it is unclear whether CSEMS can be removed when using NBCA because NBCA and CSEMS may stick together.

Moreover, there are some precautions for CSEMS placement. First, holding the guidewire in the bile duct is important when bleeding during ERCP. If the guidewire comes out of the bile duct, it may not be possible to place it again since the endoscopic visual field could not be secured. Second, the area of the obstructed bile duct and the hepatic volume that can be drained must be predicted because placing CSEMS in the perihilar bile duct causes obstruction of the contralateral bile duct. There are reports that drainage of ≥50% of the liver volume is required to have no obstructive jaundice and that drainage of ≥33% of the liver volume in the normal liver is required.^8^, ^9^

In this case report, covered SEMS was placed in the right bile duct stent; however, the left hepatic duct was not obstructed due to the side‐by‐side placement of the uncovered SEMS. Even if the posterior segment was obstructed by CSEMS and drainage for the posterior segment by CSEMS or plastic stent was achieved, PTBD was unnecessary.

In conclusion, performing IVR is the most effective method to control bleeding from a pseudoaneurysm in the perihilar bile duct; however, if severe bleeding causes changes in the hemodynamics, CSEMS placement may be useful as an alternative if IVR is not possible or available in facilities.

## CONFLICT OF INTEREST

The authors declare that they have no conflict of interest.

## FUNDING INFORMATION

None.

## ETHICS STATEMENT

All procedures were performed according to the revised declaration of Helsinki.
